# Redox coupling of lactate and β-hydroxybutyrate: An inter-organ circuit linking metabolic flexibility, mitochondrial adaptation, and disease

**DOI:** 10.1016/j.redox.2026.104279

**Published:** 2026-06-26

**Authors:** Donghai Lin, Xu Qiu, Yanan Wang, Yang Xiang, Caihua Huang

**Affiliations:** a Key Laboratory for Chemical Biology of Fujian Province, College of Chemistry and Chemical Engineering, Xiamen University, Xiamen, Fujian, 361005, China; b Key Laboratory of Marine Genetic Resources, Third Institute of Oceanography, Ministry of Natural Resources, Xiamen, Fujian, 361005, China; c Med-X Institute, Center for Immunological and Metabolic Diseases, and Dept. of Endocrinology, First Affiliated Hospital of Xi'an Jiaotong University, Xi'an Jiaotong University, Xi'an, Shaanxi, 710061, China; d Metabolic Control and Aging, Human Aging Research Institute and School of Life Science, Nanchang University, Jiangxi Key Laboratory of Human Aging, Nanchang, Jiangx, 330031, China; e Research and Communication Center of Exercise and Health, Xiamen University of Technology, Xiamen, China

**Keywords:** β-hydroxybutyrate, Lactate, Redox coupling, Inter-organ metabolism, Metabolic flexibility, Redox signaling

## Abstract

Lactate and β-hydroxybutyrate (βHB), once regarded mainly as metabolic byproducts or alternative fuels, are now increasingly recognized as redox-active metabolites that regulate energy partitioning, mitochondrial function, and adaptive stress responses. Here, we propose a unifying framework in which lactate and βHB form a redox-coupled inter-organ circuit linking the liver, kidney, heart, and skeletal muscle. Through coordinated LDH- and BDH1-dependent reactions and monocarboxylate transport, the lactate–βHB axis integrates carbohydrate and lipid metabolism, supports dynamic fuel switching, and links distinct cytosolic and mitochondrial NAD^+^/NADH redox states during fasting, exercise, hypoxia, and metabolic stress. Disruption of this circuit contributes to mitochondrial dysfunction, impaired metabolic flexibility, and maladaptive redox signaling in disorders including metabolic dysfunction-associated steatotic liver disease, type 2 diabetes, chronic kidney disease, heart failure, and sarcopenia. Beyond their bioenergetic roles, lactate and βHB also act as signaling metabolites that influence transcriptional, epigenetic, post-translational, and stress-response pathways, including protein lysine lactylation and β-hydroxybutyrylation, thereby linking metabolic state to cellular adaptation, tissue resilience, and long-term remodeling. Importantly, interventions including exercise, ketogenic or low-carbohydrate diets, SGLT2 inhibition, ketone-based strategies, and NAD^+^-enhancing approaches may help restore lactate–βHB coupling and improve redox homeostasis. This framework positions the lactate–βHB axis as a systems-level mechanism of inter-organ redox communication and provides a redox-biological basis for therapeutic targeting in metabolic and degenerative disease.

## Highlights

• Lactate and βHB form a redox-linked inter-organ circuit across metabolic tissues.
• LDH- and BDH1-dependent reactions connect distinct cytosolic and mitochondrial redox pools.
• The lactate–βHB axis supports substrate switching, mitochondrial adaptation, and stress resilience.
• Disruption of this circuit contributes to metabolic inflexibility and maladaptive redox signaling.
• Exercise, dietary, ketone-based, and NAD^+^-enhancing strategies may restore redox homeostasis.

## 1 Introduction

Lactate and β-hydroxybutyrate (βHB), once regarded mainly as metabolic byproducts or alternative fuels, are now increasingly recognized as redox-active metabolites that connect substrate availability, mitochondrial function, and adaptive stress responses across metabolically active tissues. βHB, generated mainly during fasting, carbohydrate restriction, prolonged exercise, or SGLT2 inhibition, provides an efficient oxidative substrate and participates in transcriptional, epigenetic, and antioxidant regulation [1]. Lactate, produced through glycolysis and exchanged among tissues through monocarboxylate transporters, functions not only as a rapid energy substrate and gluconeogenic precursor, but also as a practical indicator and modulator of cytosolic redox balance [2]. These features make lactate and βHB particularly relevant to metabolic flexibility, defined here as the capacity to switch among carbohydrate-, lipid-, lactate-, and ketone-based fuel use while maintaining compartment-specific redox homeostasis.

The rationale for examining lactate and βHB together is that both metabolites are linked to NAD^+^/NADH-dependent reactions, mitochondrial substrate flux, and inter-organ energy redistribution. However, the molecular mechanisms underlying their coupling remain incompletely elucidated. In this review, “coupling” therefore refers to coordinated redox-linked regulation between distinct cytosolic and mitochondrial pools, rather than to a direct lactate-to-βHB carbon flux. LDH-dependent pyruvate/lactate interconversion primarily reports and shapes the cytosolic NAD^+^/NADH state, whereas BDH1-dependent acetoacetate/βHB interconversion primarily reflects the mitochondrial NAD^+^/NADH state in BDH1-rich tissues. These two redox pools are separated by the mitochondrial boundary and communicate through redox shuttles, transporter-dependent substrate exchange, and respiratory-chain activity rather than through direct equilibration. In parallel, lactate- and βHB-derived lysine acylations, including lactylation and β-hydroxybutyrylation, broaden this axis from a fuel/redox network to a regulatory system that can affect gene expression, protein function, inflammation, and stress adaptation [3,4].

This framework is clinically relevant because disruption of lactate and βHB handling is associated with several diseases characterized by impaired metabolic flexibility and mitochondrial adaptation. In metabolic dysfunction-associated steatotic liver disease (MASLD) and type 2 diabetes (T2D), altered hepatic substrate partitioning, lactate accumulation, and impaired ketogenesis contribute to mitochondrial dysfunction and redox imbalance [5,6]. In chronic kidney disease (CKD), defective βHB handling, lactate retention, tubular hypoxia, and acid-base disturbance weaken renal redox buffering [7,8]. In heart failure (HF), the myocardium undergoes maladaptive changes in lactate, fatty-acid, glucose, and ketone-body oxidation, contributing to impaired energetic efficiency and mitochondrial stress [9,10]. In sarcopenia and insulin resistance, reduced muscle oxidative capacity and altered lactate–βHB exchange contribute to metabolic inflexibility and muscle dysfunction [11]. Thus, these disorders should not be interpreted only as isolated organ diseases, but also as failures of cross-organ redox coordination.

On this basis, the present review advances a systematic redox-centered framework in which lactate and βHB operate as a coupled inter-organ circuit linking the liver, kidney, heart, and skeletal muscle. The review is organized to address four related questions: how lactate and βHB are produced, transported, and oxidized across organs; how LDH- and BDH1-dependent reactions connect but do not merge cytosolic and mitochondrial NAD^+^/NADH pools; how disruption of this axis contributes to MASLD, T2D, CKD, HF, sarcopenia, and related metabolic states; and how interventions such as exercise, dietary modulation, SGLT2 inhibition, ketone-based strategies, and NAD^+^-enhancing approaches may act at different entry points of the same redox network. By emphasizing metabolic flexibility and mitochondrial adaptation, this review aims to move beyond separate descriptions of lactate or ketone metabolism and provide an integrated basis for interpreting disease mechanisms and therapeutic opportunities.

## 2 Liver: hub of ketogenesis and lactate recycling

**Fig. 1:**
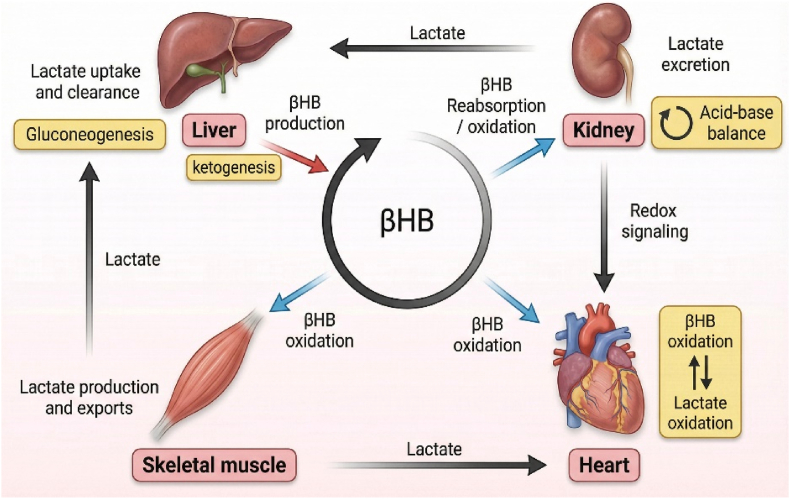
**Inter-organ coordination of lactate and β-hydroxybutyrate metabolism.** This schematic illustrates how the liver, kidney, heart, and skeletal muscle exchange lactate and βHB to maintain systemic energy and redox homeostasis. The liver recycles lactate through gluconeogenesis and generates βHB through ketogenesis. The kidney reabsorbs and oxidizes βHB while regulating lactate excretion to support acid–base balance. The heart and skeletal muscle oxidize both substrates to sustain ATP production. Together, these processes form an integrated inter-organ circuit that supports metabolic flexibility and redox communication.

The liver serves as the central metabolic and redox hub of the lactate-βHB axis, integrating carbohydrate and lipid metabolism through lactate clearance, gluconeogenesis, glycogen synthesis, and ketone-body production (Fig. 1). Through coordinated LDH- and BDH1-dependent reactions, hepatocytes place the cytosolic pyruvate/lactate redox couple and mitochondrial acetoacetate/βHB redox couple within the same metabolic network; however, these processes should not be interpreted as a strong direct substrate coupling between lactate oxidation and ketogenesis. Rather, hepatic lactate-derived pyruvate is used predominantly for gluconeogenesis during fasting and for glycogen synthesis in the postprandial state, whereas ketogenesis is driven mainly by fatty-acid β-oxidation, acetyl-CoA supply, and oxaloacetate diversion from the tricarboxylic acid (TCA) cycle. These processes influence distinct cytosolic and mitochondrial NAD^+^/NADH pools rather than a single unified NAD^+^/NADH balance, supporting mitochondrial function and preserving metabolic flexibility (Table 1). These two redox pools differ markedly in their steady-state reduction level: in well-fed rat liver, the free cytosolic [NAD^+^]/[NADH] ratio is approximately 725, whereas the mitochondrial [NAD^+^]/[NADH] ratio is approximately 8, indicating an approximately 100-fold redox gradient between compartments [12]. In BDH1-rich tissues such as liver, the mitochondrial reaction acetoacetate + NADH + H^+^ ⇌ βHB + NAD^+^ is close to equilibrium; thus, the acetoacetate/βHB ratio provides a useful index of mitochondrial [NAD^+^]/[NADH], whereas an increase in βHB relative to acetoacetate lowers this ratio and indicates a more reduced mitochondrial matrix redox state [12,13]. This hepatic redox integration is essential for adaptive fuel redistribution during fasting, exercise, and metabolic stress. When disrupted in conditions such as MASLD, T2D, and HF, this coupling impairs βHB synthesis, limits lactate disposal, and promotes mitochondrial dysfunction, redox imbalance, and reduced adaptive capacity [5,9,14]. Conversely, interventions such as exercise, caloric restriction, ketogenic diets, SGLT2 inhibition, and NAD^+^-enhancing strategies may help restore hepatic redox homeostasis, improve substrate switching, and reinforce the liver's role in systemic metabolic resilience.

**Table 1:** Comparative Roles of Lactate and β-Hydroxybutyrate in Energy Metabolism and Redox Regulation Across Metabolically Active Organs.

Organ	Lactate: Metabolic and Redox Role	βHB: Metabolic and Redox Role	Shared Redox-Coupled Features
**Liver (ketogenic and gluconeogenic hub)**	Oxidized to pyruvate via LDH to support gluconeogenesis during fasting and glycogen synthesis after feeding; the pyruvate/lactate ratio provides a practical index of cytosolic [NAD^+^]/[NADH] in LDH-rich liver tissue.	Synthesized mainly from fatty acid-derived acetyl-CoA through ketogenesis; the acetoacetate/βHB ratio reflects mitochondrial [NAD^+^]/[NADH] in BDH1-rich liver mitochondria, and βHB is exported as an energy and redox-active metabolite.	LDH- and BDH1-dependent reactions link, but do not directly equilibrate, cytosolic and mitochondrial NAD^+^/NADH pools; this coordination is shaped by MCTs, redox shuttles, fatty-acid oxidation, gluconeogenic demand, and PPARα-related metabolic regulation.
**Kidney (acid–base and redox buffer)**	Taken up and converted to pyruvate to support gluconeogenesis and oxidative metabolism in the renal cortex; lactate handling and excretion also contribute to acid–base regulation and cytosolic redox balance.	Taken up from the circulation through MCTs/SMCTs and oxidized for ATP generation; BDH1-mediated βHB oxidation generates mitochondrial NADH and contributes to renal energy metabolism and redox buffering.	Coordinated lactate handling and βHB oxidation integrate energy metabolism, NAD^+^/NADH interconversion, and pH homeostasis through shared transporters, regional oxygen gradients, and redox enzymes.
**Heart (high-efficiency oxidative organ)**	Rapidly oxidized via LDH–PDH-dependent pathways during exercise or energetic stress; supports cytosolic NAD^+^/NADH interconversion and oxygen-efficient ATP production.	Serves as an efficient mitochondrial fuel during fasting, diabetes, or heart failure; BDH1-mediated βHB oxidation generates mitochondrial NADH and supports oxidative metabolism, with context-dependent effects on ROS production.	Substrate flexibility mediated by MCT1/2, LDH, BDH1, PDH, and mitochondrial oxidative capacity enables dynamic redox-adaptive fuel switching between lactate, βHB, glucose, and fatty acids.
**Skeletal Muscle (lactate shuttle and adaptive sink)**	Produced during glycolysis and exported via MCT4, particularly in fast-twitch fibers; also functions as a shuttle fuel for oxidative tissues and contributes to cytosolic redox balance during changing energetic demand.	Imported via MCT1 and oxidized during fasting, carbohydrate restriction, or endurance exercise; βHB oxidation generates mitochondrial NADH and contributes to oxidative metabolism and redox-sensitive signaling.	Exercise-induced regulation of MCT1/4, LDH, BDH1, mitochondrial enzymes, and oxidative capacity supports metabolic flexibility, NAD^+^/NADH interconversion, redox balance, and mitochondrial adaptation.

### 2.1 Energy metabolism: integrated coordination of ketogenesis and the cori cycle

The liver preserves systemic metabolic flexibility by coordinating lactate recycling with ketone-body production during fasting, prolonged exercise, and carbohydrate restriction. However, these processes are metabolically coordinated rather than directly coupled through a major lactate-to-ketone carbon flux. Lactate taken up through monocarboxylate transporters is converted to pyruvate by LDH and used mainly for gluconeogenesis during fasting or for glycogen synthesis after feeding. In contrast, ketogenesis is driven primarily by fatty-acid β-oxidation, mitochondrial acetyl-CoA accumulation, and reduced TCA-cycle entry when gluconeogenic demand withdraws oxaloacetate. This promotes formation of acetoacetate and βHB, which are exported to extrahepatic oxidative tissues as efficient mitochondrial fuels [15,16].

This hepatic coordination links carbohydrate and lipid metabolism according to nutrient state and energetic demand. During fasting and other catabolic states, lactate-derived pyruvate supports glucose production, whereas fatty-acid-derived acetyl-CoA is redirected toward ketogenesis [17,18]. When βHB is oxidized to acetoacetate by BDH1 in extrahepatic tissues, it directly generates mitochondrial NADH for oxidative metabolism. Beyond its bioenergetic role, βHB also reflects hepatic nutrient status and participates in adaptive signaling programs linked to antioxidant defense and cellular stress resistance [19,20]. Thus, hepatic lactate handling and βHB production are best viewed as parallel nutrient-state responses that support systemic fuel redistribution and metabolic flexibility, while the detailed compartment-specific redox mechanisms connecting these processes are discussed below [12,13,21].

### 2.2 Interplay: indirect redox crosstalk between hepatic lactate handling and βHB production

The relationship between hepatic lactate handling and βHB production is redox-modulated but relatively weak as a direct substrate coupling. Lactate taken up via MCT1 is oxidized to pyruvate by LDH, thereby providing substrate mainly for gluconeogenesis during fasting or for glycogen synthesis in the postprandial state. During lactate-to-glucose conversion, LDH generates one cytosolic NADH from NAD^+^, whereas the GAPDH step of gluconeogenesis consumes cytosolic NADH in an approximately matched stoichiometry. Therefore, hepatic lactate uptake for gluconeogenesis is not expected to substantially alter the cytosolic NAD^+^/NADH redox state when LDH and GAPDH reactions are tightly coupled. Because hepatic LDH activity is high and the cytosolic lactate–pyruvate reaction is near equilibrium, changes in the tissue pyruvate/lactate ratio provide an experimentally useful readout of cytosolic [NAD^+^]/[NADH] [12,22]. However, cytosolic reducing pressure may increase when lactate/pyruvate balance changes disproportionately, when LDH-derived NADH is not fully counterbalanced by gluconeogenic NADH consumption, or when redox-shuttle capacity and downstream metabolic flux are altered [23].

In parallel, hepatic ketogenesis is controlled primarily by fatty-acid β-oxidation, mitochondrial acetyl-CoA availability, oxaloacetate diversion toward gluconeogenesis, and ketogenic enzyme activity rather than by lactate oxidation itself [24]. BDH1-mediated interconversion of acetoacetate and βHB reports the mitochondrial NAD^+^/NADH redox state in BDH1-rich tissues such as liver. Increased βHB relative to acetoacetate lowers the acetoacetate/βHB ratio and indicates a more reduced mitochondrial matrix [NAD^+^]/[NADH] state [12,13,21]. Thus, lactate/pyruvate and acetoacetate/βHB should be interpreted as linked but distinct redox couples: the former primarily reflects the cytosolic redox state, whereas the latter primarily reflects the mitochondrial matrix redox state.

The large difference between hepatic cytosolic and mitochondrial [NAD^+^]/[NADH] ratios means that cross-compartment redox coupling must be interpreted as shuttle-mediated communication rather than direct equilibration. The malate–aspartate shuttle (MAS) transfers cytosolic reducing equivalents into mitochondria through Aralar/Citrin-dependent glutamate–aspartate exchange and is constrained by mitochondrial energetics; therefore, mitochondrial NADH cannot simply be exported back to the cytosol through the same route [25,26]. When the cytosolic pyruvate/lactate ratio is reduced and LDH-derived reducing equivalents are not fully counterbalanced by gluconeogenic NADH consumption, increased cytosolic reducing pressure may stimulate MAS-dependent transfer of reducing equivalents into the mitochondrial matrix. This can lower mitochondrial [NAD^+^]/[NADH] and shift BDH1 toward reduction of acetoacetate to βHB, providing a plausible but limited redox route by which hepatic lactate/pyruvate status may influence βHB formation. This should not be interpreted as a major direct lactate-to-ketone pathway, because hepatic βHB production remains driven mainly by fatty-acid oxidation, acetyl-CoA supply, oxaloacetate withdrawal, and ketogenic enzyme activity.

The reverse relationship is also indirect and tissue-specific. Although a reduced mitochondrial acetoacetate/βHB ratio may influence cytosolic pyruvate/lactate status, mitochondrial reducing equivalents are not directly transported back to the cytosol through MAS. Instead, a reduced mitochondrial matrix [NAD^+^]/[NADH] may slow MDH2-dependent malate oxidation and limit MAS flux, which can secondarily decrease cytosolic NADH oxidation and favor a more reduced cytosolic [NAD^+^]/[NADH] state. This effect varies among tissues because alternative redox shuttles can dissipate cytosolic reducing equivalents. For example, cells with substantial glycerol-3-phosphate shuttle activity can transfer cytosolic reducing equivalents to mitochondria through FAD-linked mitochondrial glycerol-3-phosphate dehydrogenase, thereby buffering cytosolic NADH independently of MAS [27]. Similarly, tissues with high MDH2 activity or high Aralar/Citrin capacity may maintain MAS flux even when mitochondrial redox conditions change. In tissues with substantial PDH activity, a more reduced mitochondrial matrix can also inhibit PDH through NADH/product feedback and regulatory phosphorylation, thereby limiting pyruvate oxidation and altering mitochondrial redox balance [28].

**Fig. 2:**
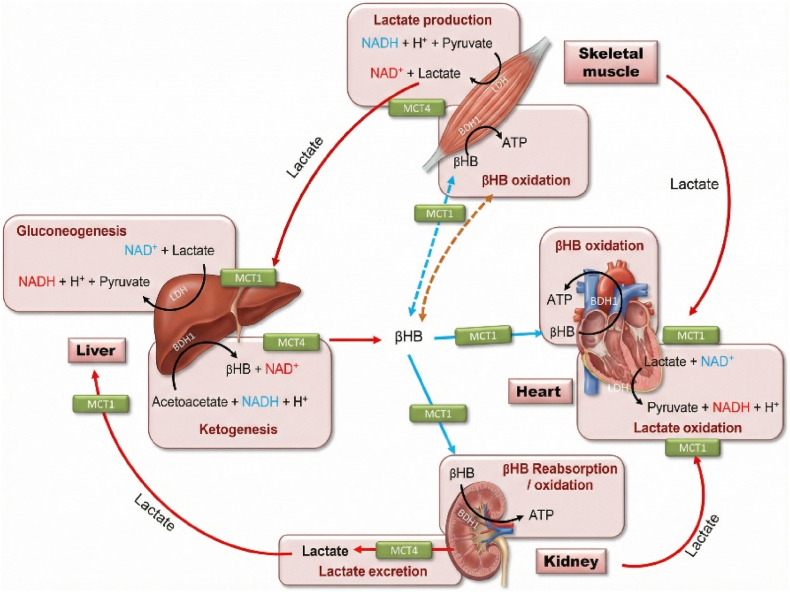
**Redox-coupled coordination of lactate and β-hydroxybutyrate metabolism across organs.** This schematic illustrates how lactate and βHB fluxes integrate liver, kidney, heart, and skeletal muscle metabolism through a shared NAD^+^/NADH-dependent network. In the liver, lactate supports gluconeogenesis, whereas ketogenesis generates βHB for export to peripheral tissues. The kidney reabsorbs and oxidizes βHB while regulating lactate handling to support acid–base balance. The heart and skeletal muscle oxidize βHB as an efficient mitochondrial fuel and interconvert lactate and pyruvate during changing energetic demand. Together, these coordinated fluxes form a redox-linked circuit that supports energy distribution, mitochondrial function, and systemic metabolic flexibility.

Thus, hepatic lactate–βHB redox coupling should be viewed as conditional, indirect, and tissue-specific rather than as a strong direct or uniformly bidirectional pathway. It depends on MAS capacity, glycerol-3-phosphate shuttle activity, MDH2 and Aralar/Citrin abundance, PDH flux, respiratory-chain activity, fatty-acid oxidation, and ketogenic demand. Concurrent fatty-acid β-oxidation provides mitochondrial acetyl-CoA for ketogenesis, allowing the liver to supply ketone bodies as complementary fuels for oxidative tissues, whereas lactate-derived pyruvate supports glucose production through gluconeogenesis (Fig. 2). In this way, hepatic lactate handling and βHB production function as coordinated but distinct components of a broader redox-buffering and fuel-redistribution system.

As summarized in Fig. 4, the lactate/pyruvate and acetoacetate/βHB couples should be interpreted as two linked but distinct redox compartments: an upper cytosolic pyruvate/lactate–[NAD^+^]/[NADH] pool and a lower mitochondrial acetoacetate/βHB–[NAD^+^]/[NADH] pool. These compartments are separated by the mitochondrial boundary and connected mainly through redox shuttle-mediated communication rather than direct equilibration. This framework makes clear that lactate/pyruvate primarily reports cytosolic redox status, whereas acetoacetate/βHB primarily reports mitochondrial matrix redox status in BDH1-rich tissues [12,13,21].

Disruption of this lactate–βHB coupling impairs NAD^+^/NADH interconversion, weakens mitochondrial redox homeostasis, and reduces metabolic flexibility in chronic metabolic disease. Conversely, interventions such as exercise, caloric restriction, and ketogenic strategies may help restore LDH–BDH1 redox alignment and reinforce hepatic mitochondrial adaptation.

### 2.3 Pathological mechanisms: Breakdown of the Lactate–βHB axis in MASLD and hepatic insulin resistance

In MASLD and T2D, disruption of the hepatic lactate–βHB axis contributes to mitochondrial dysfunction, redox disequilibrium, and insulin resistance. Chronic hyperinsulinemia suppresses ketogenesis and oxidative metabolism, shifting hepatic fuel utilization toward glycolysis and promoting lactate accumulation [6]. At the same time, during hyperinsulinemia, excess lactate delivery from skeletal muscle and adipose tissue increases hepatic redox pressure and lowers the hepatic cytoplasmic NAD^+^/NADH ratio. Because mitochondrial β-oxidation is regulated primarily within the mitochondrial compartment, this cytosolic redox shift should not be interpreted as directly constraining β-oxidation. Instead, a lower hepatic cytosolic NAD^+^/NADH ratio may influence the mitochondrial redox state through redox shuttles and may favor BDH1-mediated conversion of acetoacetate to βHB when ketogenic substrate supply is sufficient.

Lactate accumulation further promotes HIF-1α-dependent glycolytic and lipogenic reprogramming, whereas elevated acetyl-CoA and citrate stimulate de novo lipogenesis [29,30]. In parallel, suppression of PPARα signaling decreases the expression of genes required for fatty-acid oxidation and ketogenesis, thereby further disturbing βHB production and NAD^+^/NADH interconversion. Reduced βHB signaling also reduces its ability to inhibit histone deacetylases (HDACs) and thereby stimulate the expression of antioxidant and mitochondrial adaptive genes. Collectively, these changes reinforce glycolytic reprogramming, lipid accumulation, oxidative stress, and hepatic insulin resistance [23].

**Fig. 3:**
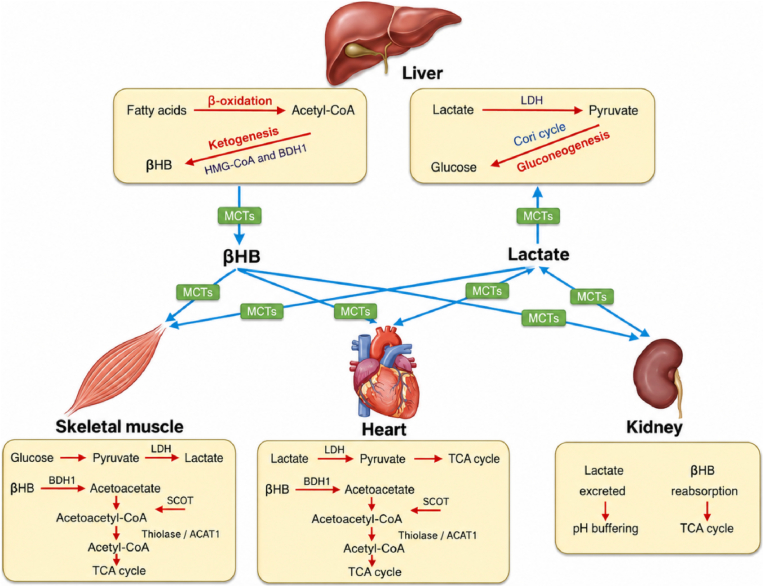
**Inter-organ redox coordination of lactate and β-hydroxybutyrate metabolism across major energy-regulating organs.** This schematic illustrates how the liver, skeletal muscle, heart, and kidney exchange lactate and βHB to sustain systemic energy and redox homeostasis. In the liver, fatty-acid β-oxidation generates mitochondrial acetyl-CoA, which feeds ketogenesis and supports production of βHB and acetoacetate, whereas lactate is converted to pyruvate by LDH and used mainly for gluconeogenesis through the Cori cycle. βHB and lactate are transported among organs through MCTs. In extrahepatic oxidative tissues such as skeletal muscle and heart, βHB is first oxidized to acetoacetate by BDH1, generating mitochondrial NADH. Acetoacetate is then activated by SCOT to acetoacetyl-CoA and subsequently cleaved by thiolase/ACAT1 to generate acetyl-CoA for TCA-cycle oxidation and ATP production. Lactate-derived pyruvate can also enter oxidative metabolism, linking cytosolic lactate/pyruvate handling with mitochondrial energy production. In the kidney, βHB handling and oxidation support renal energy metabolism, whereas lactate excretion and metabolism contribute to acid–base regulation. Together, these fluxes form a coordinated lactate–βHB circuit that supports inter-organ fuel redistribution, compartment-specific redox balance, and metabolic flexibility under physiological and pathological conditions.

Excess lactate delivery may therefore overstimulate the hepatic lactate–βHB redox circuit and contribute to a reduced cytosolic NAD^+^/NADH ratio, impaired insulin signaling, mitochondrial inefficiency, and maladaptive redox signaling, all of which are central features of MASLD and T2D. These hepatic redox disturbances may propagate across the inter-organ network and contribute to metabolic dysfunction in the heart, kidney, and skeletal muscle (Fig. 3). Conversely, interventions such as exercise, SGLT2 inhibition, ketogenic strategies, and NAD^+^-enhancing therapies may help restore hepatic redox coupling, improve βHB signaling, and reinforce mitochondrial adaptation.

**Fig. 4:**
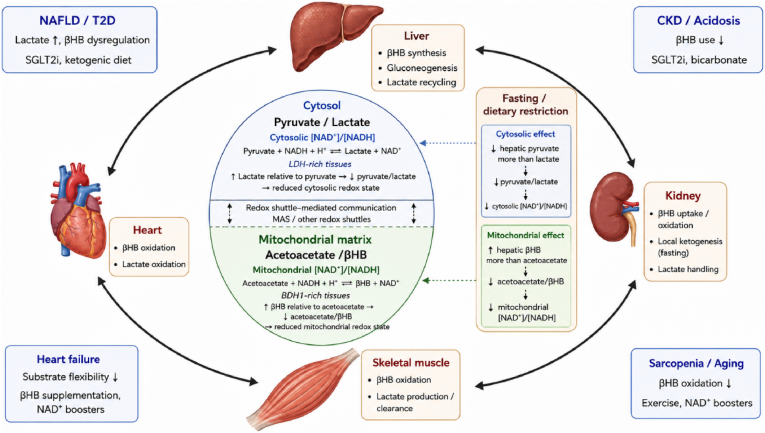
**Compartment-specific redox integration of the lactate–β-hydroxybutyrate axis in health, disease, and therapeutic restoration.** This schematic illustrates how lactate and β-hydroxybutyrate (βHB) coordinate inter-organ metabolism among the liver, kidney, heart, and skeletal muscle through linked but distinct cytosolic and mitochondrial NAD^+^/NADH redox systems. In the cytosol, the pyruvate/lactate couple reflects cytosolic [NAD^+^]/[NADH] in LDH-rich tissues, whereas in the mitochondrial matrix, the acetoacetate/βHB couple reflects mitochondrial [NAD^+^]/[NADH] in BDH1-rich tissues such as liver. Fasting or dietary restriction may decrease hepatic pyruvate more than lactate, thereby lowering the pyruvate/lactate ratio and reducing cytosolic [NAD^+^]/[NADH]. In parallel, fasting or dietary restriction increases hepatic βHB relative to acetoacetate, lowering the acetoacetate/βHB ratio and indicating a more reduced mitochondrial matrix [NAD^+^]/[NADH] state. These compartment-specific redox pools are connected mainly through redox shuttle-mediated communication rather than direct equilibration. Systemically, the liver generates βHB and recycles lactate, the heart and skeletal muscle oxidize lactate and βHB to support ATP production, and the kidney takes up and oxidizes liver-derived βHB while regulating lactate handling and acid–base balance. In disease states including NAFLD, T2D, CKD, HF, and sarcopenia, disruption of this coordination contributes to impaired βHB utilization, lactate accumulation, redox imbalance, and reduced metabolic flexibility. Interventions such as exercise, ketogenic diets, SGLT2 inhibitors, ketone-based strategies, and NAD^+^-enhancing approaches may help restore this inter-organ redox circuit and reinforce systemic metabolic resilience.

### 2.4 Clinical implications

Therapeutic strategies that target the lactate–βHB axis may help restore hepatic metabolic flexibility in MASLD and T2D (Table 2). Nutritional approaches, including ketogenic and low-carbohydrate diets, increase βHB production, enhance PPARα-related and HDAC inhibition-linked transcriptional responses, and reduce lipogenesis and inflammation, thereby improving mitochondrial function and systemic metabolic health [[31], [32], [33]].

**Table 2:** Therapeutic modulation of the lactate–β-hydroxybutyrate axis across organs.

Organ/System	Pathological Condition	Dysregulation of Lactate–βHB Axis	Therapeutic interventions
**Liver**	NAFLD	↓βHB production due to impaired ketogenesis; ↑lactate accumulation from glycolytic overload; disturbed redox balance.	Ketogenic diet; PPARα agonists; HDAC inhibitors.
**Kidney**	CKD	↓*β*HB reabsorption; ↑lactate excretion; impaired redox buffering.	SGLT2 inhibitors; *β*HB supplementation.
**Heart**	HF	↓*β*HB and lactate oxidation; reduced ATP efficiency; impaired mitochondrial redox balance.	SGLT2 inhibitors; ketone esters; PDH activators (e.g. dichloroacetate).
**Skeletal Muscle**	Sarcopenia and insulin resistance.	↓lactate export; ↓βHB oxidation; reduced mitochondrial flexibility.	Exercise; *β*HB supplementation; AMPK activators.
**Systemic/Multiorgan**	T2D; metabolic syndrome.	Global redox imbalance; ↑lactate/pyruvate ratio; ↓βHB synthesis.	Exercise, SGLT2 inhibitors, caloric restriction, redox-modulating therapy.

Pharmacological approaches provide complementary benefits. SGLT2 inhibitors induce mild ketonemia, promote fatty-acid oxidation, and alleviate hepatic steatosis while improving redox balance [34,35]. NAD^+^ precursors and pyruvate dehydrogenase activators such as dichloroacetate should be interpreted through distinct redox mechanisms. Dichloroacetate can stimulate PDH-dependent pyruvate oxidation and mitochondrial NADH generation, thereby tending to lower the mitochondrial NAD^+^/NADH ratio; consequently, its effects on lactate flux, βHB handling, and mitochondrial redox coupling are context-dependent rather than simply restorative [36].

Collectively, these interventions help normalize hepatic NAD^+^/NADH balance, improve mitochondrial efficiency, and strengthen the coordination of lactate clearance with βHB metabolism. Restoration of the lactate–βHB circuit may reduce redox stress and improve insulin sensitivity, underscoring its clinical relevance in the compartment-separated cytosolic/mitochondrial redox framework summarized in Fig. 4.

## 3 Kidney: regulator of βHB uptake, local ketogenesis, and lactate handling

Within the lactate–βHB axis, the kidney functions as a key regulator of systemic redox, metabolic, and acid–base homeostasis. By taking up and oxidizing liver-derived circulating βHB while modulating lactate clearance and release, the kidney serves as an essential component of the inter-organ redox network linking hepatic ketogenesis, skeletal-muscle lactate production, and extrahepatic oxidative metabolism [37,38] (Fig. 3; Table 3). In addition, renal proximal tubular cells can generate ketone bodies locally during fasting for intrarenal use, although renal ketogenesis is not considered a major source of circulating ketone bodies. This role is supported by marked regional compartmentalization: the renal cortex preferentially oxidizes βHB and lactate to sustain ATP production and gluconeogenic function, whereas the relatively hypoxic medulla relies more heavily on glycolysis and lactate release, thereby contributing to pH buffering and metabolic adaptation. In contrast to the liver, which generates βHB for systemic export and recycles lactate, the kidney primarily consumes liver-derived βHB, may generate ketone bodies locally for its own use during fasting, and regulates lactate flux, helping preserve NAD^+^/NADH balance, mitochondrial function, and whole-body metabolic flexibility during fasting and metabolic stress.

**Table 3:** Integrated Metabolic Pathways and Inter-Organ Cycling of Lactate and β-Hydroxybutyrate.

Process	Lactate	*β*HB
**Synthesis**	Produced from pyruvate via LDH during anaerobic glycolysis in skeletal muscle and erythrocytes.	Produced in the liver from fatty acid-derived acetyl-CoA through ketogenesis (HMG-CoA synthase and lyase).
**Transport**	Exported by MCT4 from glycolytic tissues and imported via MCT1 into oxidative tissues and the liver.	Exported from the liver and taken up by extrahepatic tissues through MCT1 and SMCT1/2 for oxidation.
**Transformation**	Converted to pyruvate by LDH, then to acetyl-CoA by PDH for entry into the TCA cycle or to glucose through gluconeogenesis.	Oxidized to acetoacetate by BDH1, then to acetyl-CoA via SCOT for oxidation in the TCA cycle.
**Oxidation**	Serves as a rapid, oxygen-efficient substrate for ATP generation in heart and skeletal muscle.	Functions as a high-efficiency mitochondrial fuel, yielding more ATP per O_2_ molecule than fatty acids.
**Utilization**	Provides immediate energy, contributes to cytosolic NAD^+^/NADH interconversion, and supports redox buffering during stress.	Sustains ATP synthesis, spares amino acids, and modulates signaling via HDAC inhibition and FOX03a/Nrf2 activation.
**Clearance**	Recycled to glucose in the liver or oxidized in heart and muscle, excess cleared renally.	Utilized by heart, skeletal muscle, and kidney; minimal urinary loss except in ketoacidosis.
**Inter-Organ Cycling**	Exported from skeletal muscle and utilized by liver (gluconeogenesis), heart, and oxidative fibers.	Synthesized in the liver and oxidized in skeletal muscle, heart, and kidney; not metabolized by the liver.

In diabetes and CKD, impaired βHB oxidation and defective lactate handling disrupt this renal redox coordination, promoting lactate retention, metabolic acidosis, mitochondrial dysfunction, and secondary disturbances in hepatic and cardiac energy metabolism (Fig. 3). Conversely, interventions such as SGLT2 inhibition, bicarbonate supplementation, and nutritional ketosis may help restore renal βHB utilization, facilitate lactate clearance, and improve NAD^+^/NADH interconversion and acid–base balance, thereby reinforcing renal redox homeostasis [39] (Table 2).

### 3.1 Energy metabolism: renal oxidation of βHB and lactate handling

The kidney contributes to systemic metabolic adaptation through coordinated oxidation of βHB, local ketone-body metabolism, and region-specific handling of lactate, particularly during fasting, prolonged exercise, and carbohydrate restriction (Fig. 3; Table 3). Renal cortical cells express MCT1 and sodium-coupled monocarboxylate transporters (SMCT1/2), which facilitate monocarboxylate uptake and tubular reabsorption of liver-derived circulating ketone bodies in proximal tubular cells. In addition, proximal tubular cells can perform limited ketogenesis during fasting for local renal use, but this locally produced ketone pool is not thought to be released substantially into the bloodstream. Once taken up from the circulation or generated locally, βHB can be oxidized by BDH1 to acetoacetate, generating mitochondrial NADH, and subsequently metabolized through SCOT-dependent pathways, underscoring the high ketolytic and oxidative capacity of the renal cortex [7,40]. This pathway enables the kidney to use ketone bodies efficiently as mitochondrial fuel under conditions of limited glucose availability while supporting renal ATP production and redox balance. In addition, GLUT2 should be interpreted as a glucose transporter in the kidney, supporting basolateral glucose movement associated with renal gluconeogenesis and tubular glucose handling rather than monocarboxylate transport.

In parallel, the kidney regulates lactate metabolism in a compartment-specific manner. Lactate can be taken up and converted to pyruvate by LDH, thereby supplying substrate for gluconeogenesis and, under oxidative conditions, for ATP generation. Through this dual function, lactate supports both renal energy production and systemic glucose homeostasis during metabolic stress [41,42]. The relative contribution of lactate oxidation, gluconeogenesis, and lactate release depends on regional oxygen tension, substrate availability, and acid–base status, with the cortex favoring oxidative metabolism and the more hypoxic medulla relying more heavily on glycolytic flux.

Together, βHB oxidation and lactate-derived pyruvate metabolism form a coordinated renal substrate system that supports mitochondrial function, NAD^+^/NADH balance, and adaptive fuel utilization. This redox-linked metabolic flexibility is especially important during sustained nutrient deprivation, when renal energy demand remains high and maintenance of tubular function depends on efficient integration of oxidative metabolism, gluconeogenesis, and acid–base regulation [8].

### 3.2 Interplay: redox coupling of βHB and lactate in renal Acid–Base regulation

The kidney supports acid–base homeostasis through coordinated handling of βHB and lactate across the cortex and medulla. During fasting or metabolic acidosis, liver-derived circulating βHB is taken up and oxidized in proximal tubular cells; in parallel, proximal tubular cells may also generate a local ketone pool during fasting for intrarenal use. In both cases, BDH1/SCOT-dependent βHB metabolism supports mitochondrial ATP production by generating mitochondrial NADH during βHB oxidation to acetoacetate and contributes to bicarbonate conservation [41,43,44]. In parallel, lactate is handled in a region-specific manner: in the cortex, lactate can be taken up and converted to pyruvate to support gluconeogenesis and bicarbonate generation, whereas in the relatively hypoxic medulla, glycolytic metabolism favors lactate production and export (Fig. 3; Table 3).

This compartmentalized substrate handling forms a redox-linked buffering system that integrates mitochondrial metabolism with pH regulation. By coupling proximal tubular ketone oxidation to regional lactate flux, the kidney coordinates NAD^+^/NADH balance, oxidative metabolism, and acid–base adaptation under metabolic stress. Under hypoxia or worsening acidosis, HIF-1α drives metabolic reprogramming toward glycolysis and lactate export, whereas reduced mitochondrial oxidation limits βHB utilization and weakens redox buffering capacity [8,45]. In CKD and DN, these adaptive responses become progressively impaired, leading to lactate retention, reduced bicarbonate generation, reduced cytoplasmic [NAD^+^]/[NADH], mitochondrial dysfunction, and persistent acidosis [42].

Thus, renal lactate–βHB coupling represents an integrated redox-buffering mechanism that links mitochondrial function, regional lactate handling, and acid–base control to preserve tubular integrity under metabolic stress.

### 3.3 Pathological mechanisms: impaired Lactate–βHB coupling in DN

In DN and CKD, progressive disruption of renal lactate–βHB coupling reflects declining metabolic flexibility and worsening redox disequilibrium. In early diabetes, increased hepatic ketogenesis elevates circulating βHB, which is normally taken up and oxidized by the kidney to support mitochondrial ATP production and redox balance [46,47]. During fasting, proximal tubular cells may also generate ketone bodies locally for their own use, but this local pathway does not represent a major endocrine source of circulating ketone bodies. As DN progresses, tubular injury and mitochondrial dysfunction impair βHB uptake and oxidation, thereby reducing ketone-supported oxidative metabolism and promoting ATP depletion, oxidative stress, and ketonuria. These changes contribute to further deterioration of renal energetic and redox homeostasis.

Concurrently, renal hypoxia and HIF-1α activation drive metabolic reprogramming toward glycolysis, increasing lactate production while limiting lactate clearance. Intracellular lactate accumulation and elevation of the cytoplasmic NADH/NAD^+^ ratio promote a state of pseudohypoxia. In cells with intact mitochondrial respiration, increased cytosolic reducing equivalents may be transferred into mitochondria through the MAS and can support oxidative phosphorylation; however, in DN and CKD, hypoxia, mitochondrial injury, and impaired respiratory-chain capacity limit this compensatory route, thereby favoring electron-pressure buildup, oxidative stress, inflammatory signaling, and tubular injury [8,48]. Impaired βHB utilization and defective lactate handling therefore converge on mitochondrial dysfunction, maladaptive redox signaling, and loss of acid–base stability, thereby reinforcing disease progression.

Therapeutic interventions such as SGLT2 inhibitors may partially restore this disrupted renal redox circuit by increasing βHB availability, improving mitochondrial resilience, and normalizing acid–base balance [49]. Enhanced βHB oxidation and improved lactate clearance may reduce redox stress, support mitochondrial NADH generation during BDH1-dependent βHB oxidation, and help preserve tubular metabolism. Preservation of the lactate–βHB axis therefore links mitochondrial integrity to renal metabolic adaptation and identifies this redox-coupled pathway as a mechanistically relevant target for slowing the progression of diabetic nephropathy.

### 3.4 Clinical implications

Therapeutic strategies that improve lactate–βHB coupling may help restore renal redox homeostasis and metabolic flexibility in DN and CKD (Table 2). SGLT2 inhibitors provide the most directly relevant example, as they increase circulating βHB, promote fatty-acid and ketone oxidation, and reduce tubular hypoxia and oxidative stress, thereby helping preserve mitochondrial function and renal energetic stability [50,51]. Enhanced βHB utilization may also support mitochondrial NADH generation during BDH1-dependent βHB oxidation, improve oxidative metabolism, and limit intracellular acidosis, although a decreased mitochondrial [NAD^+^]/[NADH] ratio from BDH1-mediated NADH generation can sometimes increase electron transport chain superoxide generation.

Nutritional interventions, including ketogenic or low-carbohydrate diets, may similarly increase βHB availability and improve renal oxidative efficiency, whereas NAD^+^-enhancing compounds may further strengthen redox coupling and coordinated lactate uptake and βHB metabolism [18]. Together, these approaches may reduce lactate accumulation, improve acid–base balance, and preserve renal oxidative capacity under metabolic stress.

Collectively, these interventions may help re-establish the renal lactate–βHB redox circuit, alleviate maladaptive redox stress, and provide a mechanistic basis for therapeutic targeting in kidney disease (Fig. 4).

## 4 Heart: coordinator of lactate and βHB oxidation for redox flexibility

The heart functions as a highly oxidative component of the lactate–βHB axis, sustaining its continuous energetic demand through coordinated utilization of lactate and βHB within an inter-organ redox network linking the liver, kidney, and skeletal muscle (Fig. 3; Table 3). By dynamically adjusting substrate preference to nutrient availability, oxygen supply, and energetic stress, cardiomyocytes use this circuit to maintain ATP production, preserve mitochondrial function, and stabilize cellular redox balance across fasting, exercise, ischemia, and heart failure [10,52].

This adaptive capacity depends on tightly coordinated transport and redox-dependent metabolism. MCT1/2-mediated substrate uptake, together with LDH- and BDH1-dependent reactions, enables rapid switching between lactate- and βHB-supported oxidative metabolism under changing physiological and pathological conditions. During acute stress, lactate oxidation can rapidly sustain ATP turnover, whereas during prolonged energetic stress, βHB utilization can provide an efficient mitochondrial substrate, generate mitochondrial NADH during BDH1-dependent oxidation, and influence reactive oxygen species (ROS) production in a context-dependent manner. When this fuel-switching capacity is disrupted, cardiac redox homeostasis deteriorates, contributing to metabolic inflexibility, mitochondrial dysfunction, and impaired myocardial energetics.

### 4.1 Energy metabolism: βHB and lactate as coordinated oxidative fuels

The heart is a highly oxidative organ with substantial substrate flexibility, dynamically shifting among fatty acids, lactate, and βHB according to physiological demand and metabolic stress. Although fatty acids predominate under resting conditions, lactate and βHB become increasingly important during exercise, fasting, diabetes, and heart failure because of their favorable oxygen efficiency and redox properties [[53], [54], [55], [56], [57]]. These adaptive shifts integrate the heart into the systemic lactate–βHB redox network and support maintenance of myocardial ATP production under changing energetic conditions (Table 3).

During acute stress or intense exercise, lactate becomes a major myocardial substrate. Increased MCT1-mediated uptake channels lactate-derived pyruvate into the TCA cycle, thereby supporting rapid ATP production with high oxygen efficiency [58]. In endurance-trained states, lactate can supply a substantial fraction of cardiac oxidative metabolism and help sustain myocardial performance under ischemic or high-demand conditions [56]. Beyond its bioenergetic role, lactate oxidation also contributes to cytosolic redox balance by facilitating NAD^+^/NADH interconversion.

βHB oxidation similarly increases during fasting, caloric restriction, diabetes, and heart failure, providing an efficient mitochondrial substrate when dependence on fatty-acid oxidation declines [57]. Following transport through MCT1/2, βHB is first oxidized to acetoacetate by BDH1, generating mitochondrial NADH; acetoacetate is then converted by SCOT to acetoacetyl-CoA and subsequently cleaved by mitochondrial thiolase/ACAT1 to yield acetyl-CoA for entry into the TCA cycle. This sequence should be distinguished from hepatic ketogenesis, in which BDH1 can operate in the opposite direction depending on mitochondrial redox state. In addition, βHB utilization may reduce oxidative stress and support mitochondrial stability during metabolic challenge [55,59]. In early heart failure, upregulation of MCT1/2 and ketolytic enzymes further enhances βHB utilization, whereas continued lactate oxidation helps preserve redox balance and oxidative flux [60,61].

Together, these substrate shifts reflect a coordinated metabolic adaptation in which lactate and βHB function as complementary oxidative fuels and redox-active metabolites. Cardiac fuel selection is therefore governed not only by substrate availability and transport capacity, but also by cytoplasmic and mitochondrial redox state, mitochondrial efficiency, and oxidative stress, allowing the heart to adapt its metabolism across physiological and pathological challenge [13,61].

### 4.2 Interplay: redox coupling and mitochondrial adaptation

Cardiac ATP production depends on coordinated oxidation of lactate and βHB, whose complementary redox properties support rapid substrate switching, mitochondrial efficiency, and energetic adaptation [52,62]. Lactate-derived pyruvate sustains TCA-cycle flux, whereas βHB oxidation by BDH1 generates mitochondrial NADH that can support oxidative phosphorylation. Through coordinated LDH- and BDH1-dependent reactions, these substrates form a redox-linked metabolic system that helps align cytosolic lactate/pyruvate handling with mitochondrial acetoacetate/βHB metabolism and fluctuating cardiac energy demand (Table 3).

This coordination also shapes mitochondrial adaptation and redox-sensitive stress signaling. By modulating NAD^+^/NADH balance, lactate and βHB influence pathways involving PGC-1α, FOXO3a, and Nrf2, thereby supporting mitochondrial biogenesis, antioxidant defense, and cellular stress tolerance [63,64]. In parallel, βHB may enhance mitochondrial stability and suppress inflammatory signaling, whereas lactate can promote oxidative efficiency and adaptive transcriptional responses, particularly during exercise or ischemic stress [[63], [64], [65]]. Preferential oxidation of lactate and βHB over fatty acids may improve ATP yield relative to oxygen consumption and help limit oxidative injury during metabolic or ischemic challenge [66,67].

When this redox coupling is disrupted, as in ischemia or heart failure, NADH/NAD^+^ imbalance, oxidative stress, and mitochondrial inefficiency increase, thereby compromising contractile performance and myocardial resilience [54,68]. Thus, intact lactate–βHB coupling should be viewed not simply as coordinated substrate oxidation, but as an integrated mechanism of cardiac redox homeostasis that sustains energy production and mitochondrial adaptation during physiological and pathological stress.

### 4.3 Pathological mechanisms: disruption of the Lactate–βHB axis in HF

HF is characterized by impaired contractility and progressive loss of substrate flexibility, limiting the myocardium's ability to switch among fatty acids, glucose, lactate, and βHB in response to energetic stress [54]. This metabolic inflexibility reduces ATP availability, disrupts redox homeostasis, and promotes oxidative stress. In the early stages of HF, declining fatty-acid oxidation is partially compensated by increased reliance on lactate and βHB, which helps preserve mitochondrial function and sustain contractile performance despite reduced oxidative efficiency.

As HF progresses, mitochondrial dysfunction, insulin resistance, and tissue hypoxia further compromise oxidative metabolism [10,69]. Reduced utilizations of lactate and βHB result in the accumulation of reducing equivalents, thereby promoting pseudohypoxia and amplifying oxidative stress and redox disequilibrium. Rising lactate levels and electron overload then damage mitochondrial components, impair oxidative phosphorylation, and accelerate energetic failure [68]. Together, these changes establish a self-reinforcing cycle of maladaptive redox signaling, metabolic inflexibility, and progressive cardiac dysfunction.

Disruption of the lactate–βHB axis therefore marks the transition from adaptive remodeling to decompensated HF. Interventions that improve redox balance, including NAD^+^-directed therapies, metabolic modulators, and mitochondrial stabilizers, may help re-establish the cardiac lactate–βHB redox circuit, restore oxidative metabolism, and alleviate maladaptive redox stress. These findings highlight this pathway as a redox-directed therapeutic target in HF.

### 4.4 Clinical implications

Therapeutic strategies that target the lactate–βHB axis may help restore cardiac redox homeostasis, metabolic flexibility, and mitochondrial function in HF (Table 2). SGLT2 inhibitors provide one of the most directly relevant examples, as they enhance myocardial βHB utilization and improve substrate efficiency, thereby supporting mitochondrial energetics under conditions of cardiac stress. Ketone-based interventions, including ketone ester supplementation, may similarly increase βHB-derived mitochondrial NADH levels during BDH1-dependent oxidation and promote oxidative metabolism, although the resulting decrease in mitochondrial [NAD^+^]/[NADH] may influence ROS generation in a context-dependent manner [70,71].

Additional approaches may complement restoration of this disrupted redox circuit, but their redox consequences should be interpreted carefully. Pyruvate dehydrogenase activators, such as dichloroacetate, may facilitate lactate-derived pyruvate oxidation; however, by increasing mitochondrial NADH generation, they would be expected to lower the mitochondrial [NAD^+^]/[NADH] ratio. This more reduced mitochondrial state may inhibit MDH2, slow MAS-dependent transfer of cytosolic reducing equivalents into mitochondria, reduce cytosolic [NAD^+^]/[NADH], and thereby limit lactate flux through LDH [72,73].

Conversely, mitochondrial pyruvate carrier (MPC) inhibitors may have the opposite effect by limiting mitochondrial pyruvate entry, increasing cytosolic pyruvate availability for LDH-dependent conversion to lactate, and thereby generating cytosolic NAD^+^ and oxidizing the cytosolic redox state. In contrast, NAD^+^ precursors, including nicotinamide riboside and nicotinamide mononucleotide, may support pyridine nucleotide availability and redox-linked energy transfer. Exercise may reinforce these effects by increasing MCT1 expression, improving lactate and βHB handling, and promoting mitochondrial biogenesis and oxidative adaptation [74,75].

Collectively, these interventions may help re-establish the cardiac lactate–βHB redox circuit, alleviate maladaptive redox stress, and provide a mechanistic basis for therapeutic targeting in heart failure (Fig. 4).

## 5 Skeletal muscle: mediator of lactate shuttling and βHB utilization

Skeletal muscle functions as both a major source and a dynamic regulator within the lactate–βHB axis, coordinating lactate release and βHB utilization as part of an inter-organ redox network that supports systemic metabolic flexibility (Fig. 3; Table 3). During anaerobic or high-intensity exertion, fast-twitch fibers export lactate via MCT4, providing a readily exchangeable substrate that can be oxidized by slow-twitch muscle, the heart, and the liver or recycled into glucose through gluconeogenesis [76,77]. Under aerobic, fasting, or carbohydrate-restricted conditions, skeletal muscle increases βHB utilization through MCT1-mediated uptake, thereby improving oxidative efficiency, supporting ATP production, and helping conserve glycogen. This dynamic substrate handling places skeletal muscle at the center of inter-organ fuel redistribution and redox coordination.

Beyond their bioenergetic roles, lactate and βHB also act as signaling metabolites that influence pathways involved in mitochondrial remodeling, antioxidant defense, and endurance adaptation. Through mechanisms involving FOXO3a, PGC-1α, and HDAC-regulated transcription, these metabolites contribute to mitochondrial biogenesis, oxidative capacity, and metabolic plasticity [78]. Disruption of this coupling is associated with maladaptive redox signaling, metabolic inflexibility, and declining muscle oxidative function in conditions such as sarcopenia, insulin resistance, and CKD. Conversely, interventions including exercise, carbohydrate restriction, and NAD^+^-enhancing strategies may help restore muscle redox homeostasis and improve substrate utilization.

### 5.1 Energy metabolism: βHB oxidation and lactate clearance during exercise

During exercise, skeletal muscle supports systemic energy production through coordinated lactate release and βHB utilization, thereby facilitating dynamic substrate exchange within the inter-organ redox network (Fig. 3; Table 3). Fast-twitch fibers export lactate during high-intensity exertion, whereas oxidative tissues, including slow-twitch muscle, the heart, and the liver, rapidly take up and oxidize lactate to sustain ATP production [79]. This lactate shuttle helps preserve glucose availability, maintain redox continuity, and support metabolic adaptation across changing workloads.

As exercise is prolonged or carbohydrate availability declines, hepatic βHB production increases, providing an oxygen-efficient mitochondrial substrate that sustains ATP synthesis, spares glycogen, and reinforces oxidative metabolism [80]. In skeletal muscle, βHB utilization follows the extrahepatic ketolytic sequence: βHB is oxidized to acetoacetate by BDH1 with mitochondrial NADH generation, acetoacetate is activated by SCOT to acetoacetyl-CoA, and acetoacetyl-CoA is cleaved by thiolase/ACAT1 to generate acetyl-CoA for TCA-cycle oxidation. By complementing lactate-supported rapid ATP turnover, βHB contributes to prolonged energy supply during sustained physiological stress. During moderate exercise, skeletal muscle coordinates the use of lactate, βHB, glucose, and fatty acids to optimize substrate selection according to energetic demand. Lactate supports rapid ATP generation and cytosolic redox balance, whereas βHB helps sustain oxidative flux and mitochondrial efficiency during prolonged workloads.

Endurance training further enhances this metabolic plasticity by increasing lactate clearance, augmenting βHB utilization, and improving mitochondrial function, thereby supporting exercise performance and counteracting metabolic decline and insulin resistance [81,82]. Through these shifts in substrate handling, skeletal muscle links peripheral energy demand to hepatic fuel production and extrahepatic oxidation [44,83]. Coordinated lactate and βHB metabolism should therefore be viewed not simply as fuel exchange, but as an adaptive mechanism of redox-linked energy redistribution during physiological stress [55].

### 5.2 Interplay: redox coupling of lactate and βHB in endurance adaptation

Skeletal muscle adapts to endurance exercise and metabolic stress through coordinated utilization of lactate and βHB, which together support ATP production, redox balance, and exercise-induced metabolic remodeling [48,76,84,85]. Isotope-tracer studies demonstrate dynamic rerouting of circulating lactate and glucose toward oxidative pathways during acute exercise, highlighting the integrated regulation of substrate selection under changing energetic demand [86]. As exercise is prolonged and glycogen availability declines, skeletal muscle shifts from predominant lactate release toward greater reliance on oxidative substrates, including βHB, in order to sustain energy supply and preserve metabolic efficiency (Fig. 2).

This coordinated substrate use depends on tight redox integration between cytosolic and mitochondrial metabolism. Lactate-derived pyruvate supports oxidative flux and contributes to cytosolic redox balance, whereas βHB oxidation through BDH1 generates mitochondrial NADH and supplies acetoacetate for SCOT- and ACAT1-dependent conversion to acetyl-CoA [87]. Beyond their roles as oxidative fuels, lactate and βHB also act as signaling metabolites that influence pathways involved in mitochondrial biogenesis, antioxidant defense, and cellular stress tolerance, thereby supporting endurance adaptation and skeletal-muscle plasticity [87]. When this coupling is impaired, oxidative capacity declines, redox homeostasis becomes destabilized, and susceptibility to muscle dysfunction and sarcopenia increases [75,88,89].

Repeated endurance exercise reinforces the lactate–βHB axis by improving lactate clearance, enhancing βHB utilization, and increasing mitochondrial content [88,90]. Through these adaptations, coordinated lactate and βHB metabolism should be viewed not simply as flexible substrate use, but as a redox-linked adaptive program that sustains oxidative performance and muscle resilience during prolonged physiological stress.

### 5.3 Pathological mechanisms: disruption of the Lactate–βHB axis in insulin resistance and sarcopenia

Disruption of the lactate–βHB axis impairs skeletal-muscle metabolism in insulin resistance and sarcopenia by weakening mitochondrial function, redox homeostasis, and metabolic flexibility. Under healthy conditions, reciprocal handling of lactate and βHB supports oxidative metabolism and adaptive substrate switching in muscle. In metabolic disease, impaired hepatic ketogenesis may reduce circulating βHB, thereby diminishing oxidative and redox support to skeletal muscle and promoting metabolic inflexibility, oxidative stress, and muscle loss [89].

Reduced βHB availability, together with defective lactate handling, disrupts mitochondrial redox balance, limits oxidative capacity and ATP production, and promotes lipid accumulation, inflammatory signaling, and insulin resistance [91,92]. These disturbances further destabilize mitochondrial function, amplify oxidative stress, and accelerate muscle dysfunction and wasting [93,94]. In this setting, impaired lactate–βHB coupling should be viewed not merely as a defect in fuel utilization, but as a broader failure of redox coordination and adaptive energy metabolism.

βHB also contributes to the preservation of muscle integrity by supporting transcriptional programs involved in mitochondrial biogenesis, antioxidant defense, and metabolic adaptation [95,96]. Loss of this signaling support further weakens lactate–βHB coupling, thereby linking insulin resistance and sarcopenia through maladaptive redox signaling, impaired mitochondrial resilience, and reduced capacity for metabolic adaptation.

### 5.4 Clinical implications

Therapeutic modulation of the lactate–βHB axis may help restore skeletal-muscle redox homeostasis, metabolic flexibility, and mitochondrial function in insulin resistance and sarcopenia (Table 2). Interventions such as endurance exercise, ketogenic or low-carbohydrate diets, SGLT2 inhibitors, and NAD^+^-enhancing therapies act in part by improving substrate switching, oxidative metabolism, and redox coordination [[97], [98], [99]].

Across these interventions, enhanced lactate clearance and increased βHB availability may improve oxidative capacity and insulin sensitivity while reducing oxidative stress and inflammatory signaling [[100], [101], [102], [103]]. Restoration of balanced lactate utilization and βHB oxidation may also preserve mitochondrial efficiency, support ATP production, and improve skeletal-muscle metabolic resilience under physiological and pathological stress.

Disruption of this coordination in metabolic disease contributes to metabolic inflexibility and mitochondrial dysfunction. Conversely, strategies that help reestablish the lactate–βHB circuit may improve muscle oxidative capacity and reinforce the translational relevance of this pathway in metabolic disease [104,105]. In this context, skeletal muscle acts as a key metabolic regulator by balancing lactate export with βHB utilization across physiological and pathological states [106] (Fig. 4).

Collectively, these strategies converge on the restoration of skeletal-muscle redox balance, mitochondrial adaptation, and metabolic plasticity. These findings identify the lactate–βHB axis as a mechanistically relevant target in insulin resistance and sarcopenia and support its therapeutic potential within a redox-biological framework (Fig. 4).

## 6 Integrated cross-organ synthesis: from organ-specific mechanisms to a redox network

The preceding sections describe the liver, kidney, heart, and skeletal muscle as major organ components of the lactate–βHB axis. However, the central value of this framework lies not in treating these organs as isolated metabolic modules, but in integrating them into a coordinated redox and substrate-exchange network. This section therefore synthesizes the organ-specific evidence into a systems-level model that links inter-organ substrate flux, compartment-specific NAD^+^/NADH regulation, disease-related metabolic inflexibility, and therapeutic modulation.

### 6.1 Cross-organ functional architecture

The lactate–βHB axis is best interpreted as an integrated systems-level network rather than as a catalogue of organ-specific pathways. In this network, each organ contributes a distinct but complementary function: the liver links lactate recycling, gluconeogenesis, fatty-acid oxidation, and ketogenesis; the kidney couples βHB uptake and oxidation with lactate handling, glucose transport, and acid–base regulation; the heart uses lactate and βHB as flexible oxidative substrates; and skeletal muscle serves as both a major lactate source and an adaptive site of βHB utilization [7,9,10,52,107]. Together, these organs form a cross-organ circuit that redistributes carbon substrates, transmits redox pressure, and supports metabolic flexibility during fasting, exercise, hypoxia, and disease.

### 6.2 Mechanistic layers of the Lactate–βHB network

The analytical value of this framework lies in distinguishing three interacting layers rather than simply listing individual pathways. The first is an inter-organ substrate-flux layer, in which skeletal-muscle lactate can be recycled by the liver through gluconeogenesis or oxidized by extrahepatic tissues, whereas liver-derived βHB and acetoacetate are exported for oxidation in heart, kidney, and skeletal muscle [7,9,44,52,107]. The second is a compartment-specific redox layer, in which the cytosolic pyruvate/lactate couple and the mitochondrial acetoacetate/βHB couple report distinct NAD^+^/NADH pools that are linked through shuttle-mediated communication rather than direct equilibration [10,23,44]. The third is an adaptive signaling layer, in which lactate and βHB influence transcriptional, epigenetic, post-translational, inflammatory, and stress-response pathways that shape mitochondrial remodeling and tissue resilience [32,33,43,48].

A key implication is that the lactate–βHB axis should be analyzed in terms of directionality, compartmentalization, and physiological context. During fasting or carbohydrate restriction, hepatic fatty-acid oxidation and oxaloacetate diversion favor ketogenesis, whereas lactate-derived pyruvate supports gluconeogenesis rather than serving as a major direct precursor for ketone-body synthesis. In contrast, during exercise or hypoxia, skeletal muscle and other glycolytic tissues increase lactate release, while oxidative tissues use lactate and βHB according to transporter expression, oxygen availability, mitochondrial capacity, and local NAD^+^/NADH state. Thus, the same metabolite change may have different meanings depending on whether it occurs in liver, kidney, heart, or skeletal muscle, and whether it reflects adaptive fuel redistribution, redox buffering, or pathological metabolic inflexibility.

**Fig. 5:**
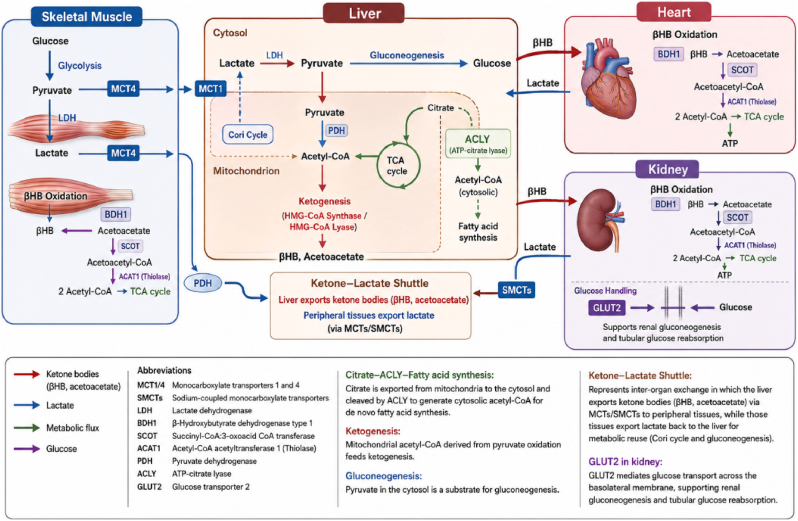
**Integrated inter-organ network linking lactate and β-hydroxybutyrate flux with redox and energy homeostasis.** This schematic illustrates how skeletal muscle, liver, heart, and kidney coordinate systemic energy distribution through lactate and βHB exchange. Skeletal muscle releases lactate through MCTs, allowing lactate to be taken up by the liver for conversion to pyruvate and subsequent use mainly in gluconeogenesis or, depending on nutritional state, oxidative metabolism. In the liver, mitochondrial acetyl-CoA derived predominantly from fatty-acid β-oxidation feeds ketogenesis through HMG-CoA synthase and HMG-CoA lyase, leading to production and export of βHB and acetoacetate. Citrate exported from the mitochondrial TCA cycle can also be converted by cytosolic ACLY to acetyl-CoA for de novo fatty-acid synthesis. The ketone–lactate shuttle shown here represents inter-organ exchange in which the liver exports ketone bodies, whereas peripheral tissues produce or exchange lactate mainly through MCTs; in the kidney, SMCTs also contribute to sodium-coupled monocarboxylate transport. This label should not be interpreted as a single direct enzymatic pathway. In extrahepatic oxidative tissues such as heart, kidney, and skeletal muscle, βHB is oxidized by BDH1 to acetoacetate, generating mitochondrial NADH; acetoacetate is then converted by SCOT to acetoacetyl-CoA and subsequently by thiolase/ACAT1 to acetyl-CoA for TCA-cycle oxidation and ATP production. In the kidney, SMCTs mediate sodium-coupled monocarboxylate transport, whereas GLUT2 supports glucose movement associated with renal gluconeogenesis and tubular glucose handling. Together, these pathways form an inter-organ metabolic and redox circuit in which lactate and βHB act as complementary energy substrates and redox-linked metabolites, thereby supporting metabolic flexibility and systemic homeostasis.

This systems-level interpretation is illustrated across the graphical framework. Fig. 3 summarizes inter-organ exchange of lactate and βHB and highlights extrahepatic ketolysis in heart and skeletal muscle. Fig. 4 provides the compartment-specific redox logic by separating the cytosolic pyruvate/lactate–[NAD^+^]/[NADH] pool from the mitochondrial acetoacetate/βHB–[NAD^+^]/[NADH] pool. Fig. 5 integrates these mechanisms into a broader metabolic network, showing how hepatic gluconeogenesis and ketogenesis, renal monocarboxylate and glucose handling, cardiac βHB/lactate oxidation, and skeletal-muscle lactate export and βHB use are coordinated through shared transporters, redox enzymes, and mitochondrial substrate flux.

### 6.3 Disease-level disruption of cross-organ redox coordination

From this perspective, metabolic diseases should be interpreted not only as local organ dysfunctions but also as failures of cross-organ redox coordination. MASLD and T2D may impair hepatic substrate partitioning and ketogenesis, CKD may disrupt renal βHB handling and lactate clearance, HF may limit cardiac substrate flexibility, and sarcopenia may reduce muscle oxidative capacity and lactate–βHB exchange [5,6,8,10,43]. These disease-specific disturbances can reinforce one another across organs, for example when hepatic redox imbalance alters circulating βHB availability, renal dysfunction impairs lactate and ketone handling, or skeletal-muscle metabolic inflexibility changes lactate delivery to liver and heart.

The disease relevance of the lactate–βHB axis therefore depends on network-level interpretation. Increased lactate, reduced βHB availability, impaired βHB oxidation, or altered acetoacetate/βHB and pyruvate/lactate ratios may have different implications according to organ context, nutritional state, oxygen availability, mitochondrial capacity, and disease stage. Such changes may represent adaptive fuel redistribution in one setting but maladaptive redox stress or metabolic inflexibility in another. This context dependence highlights the need to interpret lactate–βHB metabolism as a dynamic cross-organ circuit rather than as isolated changes in circulating metabolites.

### 6.4 Therapeutic implications and practical guidance

Therapeutically, this integrated view suggests that exercise, fasting or dietary restriction, ketogenic or low-carbohydrate diets, SGLT2 inhibition, ketone-based strategies, and NAD^+^-enhancing approaches may exert benefit by acting at different entry points of the same lactate–βHB network rather than by targeting a single organ in isolation [32,35,49,51,108]. The remaining challenge is to define when such interventions restore adaptive redox coupling and when they may exaggerate compartment-specific reductive or oxidative stress. Future work should therefore prioritize quantitative flux analysis, tissue-specific redox measurements, and intervention studies that evaluate the liver–kidney–heart–muscle circuit as an integrated system rather than as independent organ modules.

For practical guidance, these interventions should be framed according to disease context, safety considerations, and the specific component of the lactate–βHB network being targeted. For exercise, combined aerobic and resistance training provides the broadest rationale because aerobic work can enhance lactate clearance, mitochondrial oxidative capacity, and substrate flexibility, whereas resistance training helps preserve skeletal-muscle mass and metabolic reserve. Moderate-intensity continuous aerobic exercise may serve as a general foundation for MASLD, T2D, CKD, and sarcopenia, while interval or high-intensity approaches may be considered in metabolically stable individuals when the goal is to increase lactate turnover, MCT expression, and mitochondrial adaptation; in HF, advanced CKD, frailty, or severe sarcopenia, exercise intensity and progression should be individualized and clinically supervised [75,81,82,88,97,106].

For low-carbohydrate or ketogenic dietary strategies, implementation should emphasize gradual reduction of refined carbohydrates, adequate protein to preserve lean mass, nutrient-dense unsaturated fat sources, dietary fiber, hydration and electrolyte management, and monitoring of glucose, renal function, acid–base status, and medication interactions. Thus, βHB elevation should not be treated as uniformly beneficial; instead, dietary interventions should be titrated to support substrate switching and mitochondrial adaptation while avoiding excessive ketonemia, muscle loss, acid–base stress, or renal burden [[31], [32], [33],^35^,^97^,^108^].

## 7 Limitations and future perspectives

Viewing the lactate–βHB axis as an integrated inter-organ redox circuit provides a useful framework for understanding how metabolic communication is coordinated across tissues under physiological and pathological stress. However, current evidence still has several important limitations. First, many available studies examine lactate or βHB separately, focus on one organ or disease model, or infer cross-organ coupling indirectly from circulating metabolite concentrations rather than from simultaneous tissue-specific flux measurements. Second, the cytosolic pyruvate/lactate and mitochondrial acetoacetate/βHB couples provide useful redox readouts, but they do not fully capture compartment-specific NAD^+^/NADH dynamics, redox-shuttle capacity, respiratory-chain activity, or tissue heterogeneity. Third, much of the mechanistic evidence comes from animal models, cell systems, acute metabolic challenges, or selected tracer and imaging studies, whereas longitudinal human studies that integrate liver, kidney, heart, and skeletal muscle responses remain limited [7,107,109].

Several additional uncertainties should be considered when interpreting the translational value of this axis. Lactate and βHB concentrations may reflect adaptive fuel redistribution in one setting but maladaptive reductive or oxidative stress in another, depending on nutritional status, oxygen availability, disease stage, transporter expression, mitochondrial capacity, and medication use. Moreover, interventions such as fasting, ketogenic or low-carbohydrate diets, SGLT2 inhibition, ketone supplementation, exercise, and NAD^+^-enhancing approaches may not have uniform effects across organs or patient populations. Their benefits and risks may depend on whether they restore coordinated redox coupling or instead intensify compartment-specific redox imbalance, acid–base stress, or substrate overload.

Future studies should therefore move beyond single-organ or single-metabolite descriptions and combine multi-omics, isotope tracing, spatial metabolomics, real-time redox imaging, and organ-specific functional readouts to define the hierarchy, tissue specificity, and temporal dynamics of lactate–βHB-mediated redox signaling. Such approaches will be essential for clarifying how fluctuations in lactate and βHB availability reshape cytoplasmic and mitochondrial NAD^+^/NADH balance, mitochondrial adaptation, oxidative stress responses, and long-term metabolic remodeling through transcriptional, epigenetic, and post-translational mechanisms, including lysine lactylation and β-hydroxybutyrylation [3,4,109]. Particular attention should also be given to how LDH, BDH1, monocarboxylate transporters, redox shuttles, and respiratory-chain activity are integrated across organs to coordinate substrate switching and redox homeostasis.

From a therapeutic perspective, interventions that modulate this axis—including exercise mimetics, ketone supplementation, NAD^+^-restoring strategies, and other redox-targeted approaches—represent promising opportunities for improving metabolic control in cardiometabolic and age-related disorders. Progress in this field will depend on integrative cross-organ studies that link redox mechanism to disease phenotype, intervention timing, patient selection, and therapeutic response. Such efforts may establish the lactate–βHB axis not only as a conceptual model of inter-organ metabolism, but also as a mechanistic platform for the development of redox-directed metabolic therapies [108,110].

## 8 Conclusions

Lactate and βHB should be viewed not simply as metabolic fuels, but as redox-active metabolites that coordinate systemic energy balance, mitochondrial function, and metabolic flexibility across fasting, exercise, hypoxia, and related physiological and pathological states. By linking carbohydrate and lipid metabolism within compartment-specific NAD^+^/NADH-dependent frameworks, the lactate–βHB axis enables adaptive fuel redistribution and inter-organ redox communication among the liver, kidney, heart, and skeletal muscle. Within this network, the liver serves as a metabolic hub for lactate recycling, gluconeogenesis, fatty-acid oxidation, and ketogenesis; the kidney contributes to βHB uptake and oxidation, local ketone handling, lactate metabolism, glucose transport, and acid–base regulation; the heart functions as a highly oxidative consumer of lactate and βHB; and skeletal muscle acts as both a major lactate source and a dynamic site of βHB utilization (Fig. 5).

A central implication of this framework is that lactate and βHB metabolism should be interpreted through compartment-specific redox logic. The cytosolic pyruvate/lactate couple mainly reflects cytosolic [NAD^+^]/[NADH], whereas the mitochondrial acetoacetate/βHB couple mainly reflects mitochondrial [NAD^+^]/[NADH] in BDH1-rich tissues. These redox pools are linked through shuttle-mediated communication and substrate flux, but they are not freely equilibrated. Therefore, changes in lactate, pyruvate, βHB, and acetoacetate should be interpreted according to tissue context, nutritional state, mitochondrial function, redox-shuttle capacity, and disease stage rather than as simple linear indicators of whole-cell redox balance.

Beyond their bioenergetic roles, lactate and βHB also function as signaling metabolites that transmit nutrient and redox information to transcriptional, epigenetic, post-translational, and stress-response pathways, thereby linking metabolic state to cellular adaptation, tissue resilience, and long-term remodeling. Disruption of this circuit contributes to mitochondrial dysfunction, impaired substrate switching, maladaptive redox signaling, and metabolic inflexibility in disorders including MASLD, T2D, CKD, HF, and sarcopenia.

From a therapeutic perspective, interventions such as exercise, ketogenic or low-carbohydrate diets, SGLT2 inhibition, ketone-based strategies, and NAD^+^-enhancing approaches may help restore this redox-coupled network, improve mitochondrial performance, and reinforce cross-organ metabolic flexibility. The integrated lactate–βHB framework therefore provides a mechanistic basis for understanding systemic metabolic dysfunction and supports the development of redox-directed therapeutic strategies for metabolic, cardiovascular, renal, and age-related diseases.

## Ethics declarations

The authors declare that they have no conflict of interest.

## CRediT authorship contribution statement

**Donghai Lin:** Conceptualization, Funding acquisition, Supervision, Writing – original draft, Writing – review & editing. **Xu Qiu:** Conceptualization, Investigation, Visualization, Writing – original draft, Writing – review & editing. **Yanan Wang:** Conceptualization, Funding acquisition, Writing – review & editing. **Yang Xiang:** Conceptualization, Writing – review & editing. **Caihua Huang:** Conceptualization, Writing – review & editing.

## Declaration of competing interest

The authors declare that they have no conflict of interest.

## Data Availability

No data was used for the research described in the article.

## Abbreviations

βHB, β-hydroxybutyrate; HDACs, histone deacetylases; HIF-1α, hypoxia-inducible factor-1α; LDH, lactate dehydrogenase; BDH1, β-hydroxybutyrate dehydrogenase 1; MCTs, monocarboxylate transporters; T2D, type 2 diabetes; MASLD, metabolic dysfunction-associated steatotic liver disease; SGLT2, sodium-glucose cotransporter 2; SCOT, succinyl-CoA:3-oxoacid CoA transferase; ACAT1, acetyl-CoA acetyltransferase 1; Kbhb, β-hydroxybutyrylation; DNL, de novo lipogenesis; PC, pyruvate carboxylase; CKD, chronic kidney disease; DN, diabetic nephropathy; SMCTs, sodium-coupled monocarboxylate transporters; GLUT2, glucose transporter 2; ACLY, ATP-citrate lyase; ROS, reactive oxygen species; PDH, pyruvate dehydrogenase; MPC, mitochondrial pyruvate carrier; GAPDH, glyceraldehyde-3-phosphate dehydrogenase; PDK, pyruvate dehydrogenase kinase; SOD, superoxide dismutase; HIIT, high-intensity interval training; TCA, tricarboxylic acid.
